# A new lanostane-type triterpene with lipid-lowering activity from Ganoderma lucidum and an additional analogue

**DOI:** 10.3389/fchem.2025.1726447

**Published:** 2026-01-21

**Authors:** Xuesheng Hu, Lu Han, Yiying Tan, Peitong Wu, Yajing Li, Wanjie Liu, Jiaming Shen, Yishan Li, Ming Yang, Chunnan Li, Jiaming Sun

**Affiliations:** Jilin Ginseng Academy, Changchun University of Chinese Medicine, Changchun, China

**Keywords:** Ganoderma lucidum, Ganoderma triterpenes, HepG2 cell, lipid accumulation, NAFLD

## Abstract

Non-alcoholic fatty liver disease (NAFLD) represents a chronic liver disorder with widespread global prevalence, primarily attributed to hepatic lipid accumulation, oxidative stress, and inflammatory responses. In the realm of traditional Chinese medicine, *Ganoderma lucidum* is predominantly utilized for its hepatoprotective properties. The objective of this study was to isolate and identify novel bioactive compounds capable of mitigating hepatic fat accumulation. An *in vitro* steatosis model was established using oleic acid-induced HepG2 cells to evaluate the total triglyceride (TG) content across various components. Fractionation of the compounds was guided by the observed reduction in TG content, employing multiple chromatographic techniques to successfully isolate ten Ganoderma triterpenes. Structural elucidation was achieved through 1D and 2D NMR spectroscopy, supplemented by additional spectroscopic methods. This investigation led to the identification of two previously unreported lanostane-type triterpenes (1–2) alongside eight known analogues (3–10). Compound 1 and 2 exhibit structural distinctions from the other compounds, primarily in the substituents at positions C-3, C-7, and C-15, as well as in the spatial orientation of these substituents. *In vitro* experiments were conducted to assess the efficacy of various compounds in inhibiting lipid accumulation. Compound 1-5 demonstrated a significant reduction in TG levels within the OA-induced HepG2 cell model (*p* < 0.05). In comparison to the model group, Compound 1 demonstrated a moderate lipid-lowering effect, (2.11 mmol/gprot vs. 2.70 mmol/gprot, *p* < 0.003). Conversely, Compound 2 exhibited a significantly more pronounced lipid-lowering effect, (1.27 mmol/gprot vs. 2.70 mmol/gprot, *p* < 0.0001). Furthermore, when compared with the positive control drug, the lipid-lowering efficacy of Compound 2 was significantly superior to that of Compound 1. Furthermore, the application of network pharmacology, molecular docking, and molecular dynamics simulations elucidated the mechanism of action underlying the effects of methyl ganoderenic acid A(2).

## 1 Introduction

*Ganoderma lucidum* is referenced in ancient Chinese medical texts, including the “Shen Nong Ben Cao” and the “Compendium of Materia Medica,” where its tonic properties are extensively documented. Contemporary phytochemical investigations have identified that it predominantly comprises polysaccharides, triterpenes, aromatic meroterpenoids, alkaloids, and flavonoids (Wu et al., 2024). Pharmacological research has demonstrated that *G. lucidum* exhibits immunomodulatory (Li et al., 2019), antioxidant (Wang et al., 2019), anti-tumor (Zhang et al., 2024), anti-diabetic (Chen et al., 2020), and hepatoprotective properties (Ahmad et al., 2023). The fundamental pathology of non-alcoholic fatty liver disease (NAFLD) involves the excessive accumulation of lipids in the liver, resulting in steatosis, which is often accompanied by insulin resistance, oxidative stress, and inflammation (Gu et al., 2016). This condition can progress to non-alcoholic steatohepatitis (NASH), liver fibrosis, cirrhosis, and even hepatocellular carcinoma, thereby posing a significant threat to human health (Dongiovanni et al., 2021). Currently, there are no effective treatments available for NAFLD, making the identification of new candidate compounds or novel therapeutic targets a critical focus of contemporary NAFLD research. *G. lucidum* has been shown to ameliorate the progression of NAFLD through various mechanisms, including the regulation of lipid metabolism (Zhong et al., 2022), improvement of insulin resistance (Lee et al., 2020), and antioxidative effects (Soares et al., 2013). However, further investigation is required to elucidate the specific compounds responsible for these therapeutic effects. At present, multiple studies (Lagrutta et al., 2017) have reported that OA effectively induces HepG2 cells to develop an *in vitro* fatty liver model. This model reliably facilitates triglyceride accumulation in HepG2 cells *in vivo*, offering a straightforward and reproducible approach. Consequently, in this study, we employed OA to induce HepG2 cells, thereby establishing an *in vitro* model for further investigation. In this study, oleic acid (OA) was employed to induce fatty degeneration in HepG2 cells, thereby constructing a model for investigation. Utilizing an activity-oriented approach in conjunction with various chromatographic techniques, we successfully isolated and identified ten highly oxidized lanostane-type triterpenoids. The structural elucidation of these compounds was achieved through comprehensive spectroscopic analyses, including one-dimensional (1D) and two-dimensional (2D) nuclear magnetic resonance (NMR), infrared (IR) spectroscopy, ultraviolet (UV) spectroscopy, and high-resolution electrospray ionization tandem mass spectrometry (HR-ESI-MS/MS). Notably, two previously unreported lanostane-type triterpenoid compounds were discovered. The lipid-lowering effects of these compounds were assessed in OA-induced HepG2 cells. To further elucidate the potential lipid-lowering mechanisms, network pharmacology, molecular docking, and molecular dynamics simulation techniques were employed.

## 2 Materials and methods

### 2.1 General experimental procedures

The 1D and 2D NMR data were acquired using a Bruker AVANCE III 600 MHz spectrometer (Bruker, Massachusetts, United States), with CDCl_3_ and tetramethylsilane (TMS) as the internal standard. HR-ESI-MS spectra were recorded on a UHPLC-QE Orbitrap-MS/MS system (Thermo Scientific, Massachusetts, United States). IR spectra were obtained using a Thermo Scientific Nicolet iS20 FTIR with dry KBr pellets (Thermo Scientific, Massachusetts, United States), and UV spectra were measured on an Agilent 1100 UV-DAD (Agilent Technologies Inc., California, United States). Silica gel (75–100 mesh, 200–300 mesh) was sourced from Qingdao Marine Chemical Inc., China), Sephadex LH-20 (GE, Marlborough, United States), MCI (40–75 μm, H&E Co., Ltd., Beijing, China), and ODS-C18 (50 μm, YMC, Kyoto, Japan) were employed for column chromatography. All chemical reagents utilized in this study were of analytical or HPLC grade, sourced from Shanghai Aladdin Biochemical Technology Co., Ltd. (Shanghai, China). Analytical and semi-preparative HPLC were conducted using an Agilent 1,100 liquid chromatograph equipped with an Agilent ZORBAX SB-C18 column (5 μm, 4.6 mm × 150 mm; 5 μm, 9.4 mm × 250 mm). Fractions were detected using GF254 TLC, and spots were visualized by heating after treatment with 5% H_2_SO_4_ in ethanol. Oleic acid (LOT NO. O108484) and Rosuvastatin Calcium (RC, LOT NO. R129220) were procured from Shanghai Aladdin Biochemical Technology Co., Ltd. (Shanghai, China). A 4% paraformaldehyde solution (LOT NO. 24101933) was obtained from Biosharp Company (Guangdong, China). The Oil Red O Saturated Solution (LOT NO. G1260) was acquired from Beijing Solarbio Science & Technology Co., Ltd. (Beijing, China). The total protein assay kit (BCA, LOT NO. A045-4-2), total cholesterol assay kit (TC, LOT NO. A111-1-1), triglyceride assay kit (TG, LOT NO. A110-1-1), alanine aminotransferase assay kit (ALT, LOT NO. C009-2-1), and aspartate aminotransferase assay kit (AST, LOT NO. C010-2-1) were purchased from Nanjing Jiancheng Bioengineering Institute (Nanjing, China).

### 2.2 Fungal material

The dried fruiting bodies of Ganoderma lucidum (Leyss. ex Fr.) Karst utilized in this study were procured in September 2024 from Dunhua, located in Jilin Province, China. The fungal material was authenticated by Professor Hui Zhang of Changchun University of Chinese Medicine. A voucher specimen (CLZ20240910) has been deposited at the Animal Medicine Research Laboratory of the Jilin Ginseng Academy, Changchun University of Chinese Medicine, Changchun, China.

### 2.3 Extraction and isolation

The dried fruiting bodies (5 kg) were pulverized and subjected to extraction using 95% ethanol (5 L), 70% ethanol (5 L), and purified water (5 L) under conditions of heating and reflux for 3 h, respectively. The resulting filtrates were combined to yield a crude extract weighing 310 g (yield 6.2%). This crude extract was then suspended in warm water and extracted with EtOAc (v/v 1:1) until the solution became colorless to obtain the EtOAc extract (80.5 g). The EtOAc extract was subjected to column chromatography on silica gel with gradient elution of PE-EtOAc (v/v 100:1, 90:10, 70:30, 50:50) and CH_2_Cl_2_-MeOH (v/v 100:0, 40:1, 20:1, 10:1, 5:1, 1:1, 0:1) to give six fractions. Fr.4 (20.3 g) was ultimately resolved into ten distinct compounds through successive purification steps involving silica gel column chromatography, MCI column chromatography, ODS-A, and semi-preparative HPLC. A comprehensive description of the separation process is provided in the Supplementary Material (Supplementary Figure S1).

### 2.4 Activity test

#### 2.4.1 Cell culture

HepG2 cells were procured from Shanghai Fuheng Biotechnology Co., Ltd. (Shanghai, China, Catalog No. FH0076), and their proliferation was observed via light microscopy. The cells were cultured in Dulbecco’s Modified Eagle Medium (DMEM) supplemented with 10% fetal bovine serum and 1% penicillin (100 U/mL)-streptomycin (100 μg/mL), maintained in a 37 °C incubator with 5% CO2. The culture medium was refreshed every 2 days. Upon reaching 90% confluence, the cells were subcultured at a 1:2 ratio, and experiments commenced once the cells entered the logarithmic growth phase.

#### 2.4.2 Establishment of a HepG2 cell model with steatosis

A 10 mmol/L oleic acid stock solution was prepared following the method outlined in the reference (Lagrutta et al., 2017). This stock solution was subsequently diluted with DMEM medium to achieve final concentrations of 0, 0.2, 0.4, 0.6, 0.8, 1.0, and 1.2 mmol/L for OA induction solutions. After 24 h of incubation, cell proliferation activity was assessed using the CCK-8 assay. Lipid accumulation was evaluated through Oil Red O staining and measurement of intracellular TG. The optimal OA concentration for inducing steatosis in HepG2 cells was identified and established.

#### 2.4.3 Effects of *G. lucidum* fractions and compounds on cell proliferation of HepG2 cells with steatosis

The CCK-8 assay was employed to assess the effects of various compounds and fractions on the proliferation of steatotic HepG2 cells. Experimental groups included a control group (cells + culture medium), a model group (cells + OA), a treatment group (various concentrations + OA), and a positive control group (Rosuvastatin Calcium,RC + OA).

#### 2.4.4 Effects of *G. lucidum* fractions and compounds on lipid accumulation in steatotic HepG2 cells

Based on the findings from Experiment 2.4.3, the optimal concentration that did not adversely affect the growth of steatotic HepG2 cells was selected for further investigation. An experimental design was established comprising a control group (cells + culture medium), a model group (cells + OA), a drug-treated group (OA with varying concentrations of the fractions), and a positive control group (RC). The impact of each fraction and compound on lipid accumulation in steatotic HepG2 cells was assessed using Oil Red O staining, as well as measurements of intracellular TG, TC, and the ALT and AST in the cell supernatant.

#### 2.4.5 The CCK-8 assay for cell viability

Cells in the logarithmic growth phase were harvested and seeded into a 96-well plate at a density of 5,000 cells per well. Following cell adhesion, the cells were cultured for 24 h according to the experimental conditions outlined in Sections 2.4.2 and Sections 2.4.3. Subsequently, the culture medium was aspirated, and 100 μL of fresh culture medium along with 10 μL of CCK-8 solution was added to each well. The plates were incubated for 0.5–2 h, and absorbance was measured at 450 nm. Cell viability was calculated using the formula:

Cell viability = [(A_experimental group - A_blank group)/(A_control group - A_blank group)] × 100%.

#### 2.4.6 Oil red O staining and determination of TG/TC/ALT and AST

Cells in the logarithmic growth phase should be collected and seeded into a 6-well plate at a density of 5 × 10^5 cells per well. Once the cells have adhered to the substrate, establish the experimental groups as outlined in Sections 2.4.2 and Sections 2.4.4, and maintain the cultures for a duration of 24 h. Upon completion of the incubation period, aspirate the culture medium and perform three washes with 1 mL of preheated phosphate-buffered saline (PBS), each lasting 2 min. Subsequently, apply a 4% paraformaldehyde fixative for 10 min. Following fixation, thoroughly rinse the cells with distilled water and immerse them in 60% isopropanol for 5–10 s. Add 1 mL of Oil Red O working solution, ensuring the procedure is conducted in the dark, and incubate at room temperature for 20 min. Wash the cells twice with PBS. For color separation, add 1 mL of 60% isopropanol for 20 s. Perform two washes with redistilled water and examine the oil droplet staining under a microscope. The TG and TC, as well as the ALT and AST levels in the cell supernatant, should be measured according to the instructions provided with the respective kits.

### 2.5 Analysis of the molecular mechanism of compound 2 in the treatment of NAFLD using network pharmacology and molecular docking technology

#### 2.5.1 Compound 2 target acquisition for the treatment of NAFLD

Upon obtaining the SMILES notation for Compound 2 via ChemDraw, we utilized the Swiss Target Prediction tool (http://www.swisstargetprediction.ch/) to identify potential targets associated with this compound. Subsequently, using “NAFLD” as a keyword, we conducted a search for targets linked to NAFLD-related diseases across several databases, including GeneCards (https://www.genecards.org,v5.26.0), and OMIM (https://www.omim.org, Download date: 15 October2025). In the GeneCards database, we focused on disease target proteins with relevance scores exceeding the average (Relevance score ≥2.59). Additionally, we extracted disease-related targets from the Gene Map database within OMIM and integrated these with targets from the other two databases to establish a comprehensive target database pertinent to NAFLD. In conclusion, a total of 304 targets associated with NAFLD and 110 compound targets were identified. Utilizing the Venny 2.1.0 platform, we synthesized the targets associated with the identified potential active ingredients with disease-related target information. By constructing a Venn diagram, we identified common targets, which were subsequently extracted as potential targets for Compound 2 intervention in NAFLD.

#### 2.5.2 GO and KEGG pathway enrichment analysis

The intersection data of disease targets and potential targets were imported into the DAVID database to conduct Gene Ontology (GO) and Kyoto Encyclopedia of Genes and Genomes (KEGG) pathway enrichment analyses. These analyses were designed to elucidate the biological processes and signaling pathways potentially implicated in the pharmacological treatment of NAFLD. The results of the enrichment analyses were visualized on a microinformation platform, with -log10P values represented on the vertical axis.

#### 2.5.3 Molecular docking analysis

Molecular docking studies were executed utilizing AutoDock software. The three-dimensional coordinates of the active compound were sourced from ChemDraw, while the three-dimensional structural data of the core disease target were retrieved from the Protein Data Bank (PDB) database (https://www.rcsb.org, Download date: 15 October2025). The spatial configuration was validated using PyMOL software. The docked ligand was converted to pdbqt format using Sail Vina, and water molecules within the protein complex were removed using AutoDock Tools 1.5.6. Subsequently, the ligand and receptor were isolated and saved as a pdbqt file for docking analysis. Ultimately, a three-dimensional interactive visualization model was generated using PyMOL software.

#### 2.5.4 Molecular dynamics simulation

The initial conformations for molecular dynamics simulations were derived from molecular docking results. Utilizing the Gromacs simulation software, the processes encompassed energy minimization, removal of water molecules from the receptor, addition of polar hydrogen atoms, and the introduction of charges. During the simulation procedures, energy minimization of the entire system was conducted to attain optimal molecular configurations. Subsequently, the structural integrity of the compound-protein complex was evaluated over a 200 ns simulation sampling period. Molecular Dynamics was employed to establish the simulation parameters. Initially, the system’s energy was minimized using the steepest descent method for 50,000 steps, followed by constraining the positions of heavy atoms to perform NVT and NPT equilibration for an additional 50,000 steps. The system’s temperature was maintained at 300 K, and the pressure was kept at 1 atm. The evaluation of the simulation trajectories was conducted using root-mean-square deviation (RMSD), root-mean-square fluctuation (RMSF), and the protein radius of gyration (RG) as assessment metrics.

### 2.6 Statistical analysis

Statistical analyses and graphical representations were conducted utilizing GraphPad Prism 9.5 (GraphPad Software, San Diego, CA, United States). The data are expressed as mean ± standard deviation (SD). Statistical significance is denoted as follows: (* and #) for *P* < 0.05, (** and ##) for *P* < 0.01, (*** and ###) for *P* < 0.001, (**** and ####) for *P* < 0.0001, and ns for not-significant.

## 3 Results

### 3.1 Structural characterization of compounds 1 and 2

**FIGURE 1:**
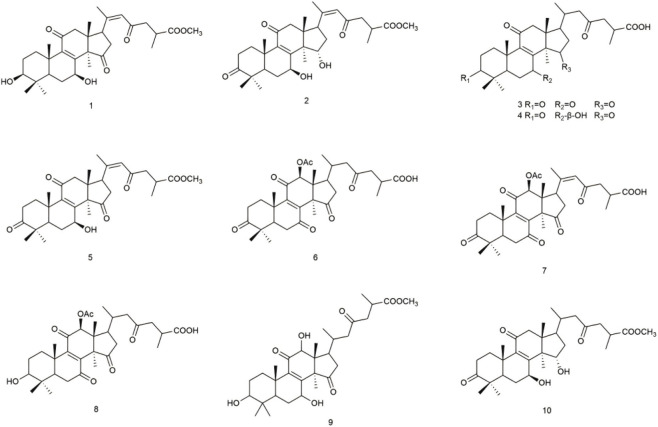
Chemical structures of compounds 1–10.

The dry fruiting bodies of *Ganoderma lucidum* (5 kg) underwent extraction with ethanol. The resultant extract was subsequently partitioned with ethyl acetate (EtOAc), yielding an EtOAc extract weighing 310 g. This extract was further fractionated using silica gel column chromatography, MCI gel, ODS-A, and semi-preparative HPLC, resulting in the isolation of 10 compounds, including two previously undescribed compounds (Figure 1).

**FIGURE 2:**
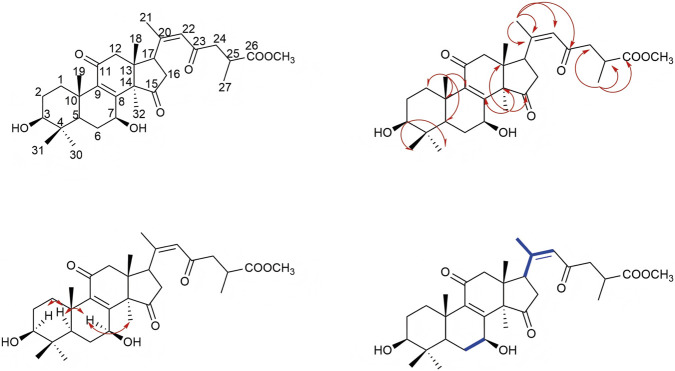
The key 2D-NMR correlations of 1.

Compound 1 was isolated as a white solid powder. HR-ESI-MS analysis revealed a peak at m/z 527.3014 [M-H]-, closely matching the theoretical value of 527.3018, which is consistent with a molecular formula of C_31_H_44_O_7_ and a degree of unsaturation of 10. IR spectroscopy indicated the presence of carbonyl and hydroxyl functional groups. The ^13^C- NMR (Table 1) and HSQC spectra identified 31 carbon signals, including three ketone carbonyl carbons (δ_C_ 216.69, 198.13, 197.20), one ester carbon (δ_C_ 176.52), and two pairs of double bond carbons (δ_C_ 156.61, 153.59, 142.91, 124.82). The compound in question comprises seven methyl carbons with chemical shifts at δ_C_ 28.32, 24.56, 21.18, 18.90, 18.52, 17.29, and 15.60, one methoxy carbon at δ_C_ 52.07, two oxygen-linked carbons at δ_C_ 78.41 and 66.99, six methylene carbons, three methine carbons, and four quaternary carbons. The presence of a carbonyl group and a double bond collectively accounts for six degrees of unsaturation. Based on the chemical shifts observed for the seven methyl hydrogens in the ^1^H-NMR spectrum (δ_H_ 1.04, 1.40, 2.15, 0.86, 1.21, 1.20, 0.85), it is preliminarily concluded that the compound exhibits a lanostane-type triterpene skeleton. The A, B, C, and D rings of this skeleton collectively contribute four degrees of unsaturation, aligning with a total of ten degrees of unsaturation. In the HMBC spectrum (Figure 2), the hydrogen atom H-27 (δ_H_ 1.20) shows correlations with carbons C-26 (δ_C_ 176.52), C-25 (δ_C_ 34.96), and C-24 (δ_C_ 47.89). The hydrogen atom associated with the methoxy group (H-COOCH3, δ_H_ 3.69) correlates with C-26 in the HMBC spectrum, suggesting that the hydrogen atom on the carboxyl group at C-26 is substituted by a methyl group. Furthermore, H-22 (δ_H_ 6.03) correlates with C-17 (δ_C_ 49.77), C-20 (δ_C_ 153.59), C-21 (δ_C_ 21.18), and C-23 (δ_C_ 198.13) in the HMBC spectrum. Similarly, in the COSY spectrum, H-22 exhibits coupling with H-21 and H-17. In the HMBC analysis, proton H-18 (δ_H_ 0.86) exhibits correlations with carbons C-12 (δ_C_ 49.21), C-13 (δ_C_ 46.38), and C-14 (δ_C_ 58.79). Similarly, proton H-19 (δ_H_ 1.21) shows correlations with carbons C-1 (δ_C_ 34.90), C-10 (δ_C_ 38.78), C-5 (δ_C_ 49.29), and C-9 (δ_C_ 142.91). Proton H-32 (δ_H_ 1.41) correlates with carbons C-13 (δ_C_ 46.38), C-14 (δ_C_ 58.79), C-8 (δ_C_ 156.61), and C-15 (δ_C_ 216.69) in the HMBC analysis. Consequently, the planar structure of this compound can be deduced as illustrated in Figure 2. A review of the literature indicates that the compound bears structural similarity to the previously reported Ganoderic acid B (Kubota et al., 1982), with notable differences including a double bond between C-20 and C-22, and the substitution of a carboxyl group at C-26 with a methoxy group. The 2D-NMR and ROESY spectra further reveal proximal spatial relationships between H-7 and H-5, as well as H-7 and H-32, corroborating that the relative configuration of the compound is β-configuration for both the 3-OH and 7-OH groups. A search in the SciFinder database indicates that this compound is previously undescribed. Therefore, the structure of compound 1 has been determined to be lanosta-8,20 (22)-dien-11,15,23-trioxo-3β,7β-diol-26-oic acid methyl ester, and it has been designated as methyl ganoderenic acid B.

**TABLE 1:** ^1^H and ^13^C NMR spectroscopic data for compounds 1–2^a^.

Position	1			2		
	δ_C, type_	δ_H_, (J in Hz)	HMBC^b^	δ_C, type_	δ_H_, (J in Hz)	HMBC^b^
1	34.90, CH_2_	2.83^c^, 0.99 m	2, 3, 9, 19	35.44, CH_2_	2.85^c^, 1.47 m	2, 3, 9, 10, 19
2	27.80, CH_2_	1.67 m, 1.65 m	1, 3, 10	34.22, CH_2_	2.50^c^	1, 3, 10
3	78.41, CH	3.22 dd (11.4, 4.9)	10, 30, 31	216.72, C	​	​
4	39.06, C	​	​	46.58, C	​	​
5	49.29, CH	0.90 m	6, 10, 30, 31	48.72, CH	1.69^c^	6, 10, 30, 31
6	26.76, CH_2_	2.20 m, 1.60 m	5, 7, 8	29.18, CH_2_	2.07 m, 1.69^c^	5, 7, 8, 9, 10
7	66.99, CH	4.83 t (8.8)	6, 8, 9	68.96, CH	4.67 days (8.5)	6, 9
8	156.61, C	​	​	158.32, C	​	​
9	142.91, C	​	​	140.39, C	​	​
10	38.78, C	​	​	38.07, C	​	​
11	197.20, C	​	​	198.62, C	​	​
12	49.21, CH_2_	2.83^c^, 2.59 days (16.3)	11, 13, 18	50.47, CH_2_	2.79 dd (15.6, 1.3), 2.38 days (15.6)	9, 11, 13, 14, 18
13	46.38, C	​	​	48.09, C	​	​
14	58.79, C	​	​	53.31, C	​	​
15	216.69, C	​	​	72.79, CH	4.91 dd (9.6, 7.3)	8, 14, 16, 20, 32
16	37.82, CH_2_	2.63 dd (9.3, 1.8)	13, 14, 17, 18, 20	32.09, CH_2_	2.44 m, 1.77 m	13, 14, 15, 17
17	49.77, CH	3.07 t (9.3)	13, 18, 21, 20, 22	52.12, CH	2.88 m	16, 18, 20, 21, 22
18	18.90, CH_3_	0.86 s	12, 13, 14	18.97, CH_3_	0.81s	12, 13, 14, 17
19	18.52, CH_3_	1.21 s	1, 5, 9, 10	19.31, CH_3_	1.27s	1, 5, 9, 10
20	153.59, C	​	​	156.35, C	​	​
21	21.18, CH_3_	2.15 s	17, 20, 22, 23	21.23, CH_3_	2.11s	17, 20, 22, 23
22	124.82, CH	6.03 s	17, 20, 21, 23	124.19, CH	6.10s	17, 20, 21, 23
23	198.13, C	​	​	198.08, C	​	​
24	47.89, CH_2_	2.96^c^, 2.53 m	23, 25, 26, 27	47.71, CH_2_	2.94^c^, 2.52 m	23, 25, 26, 27
25	34.96, CH	2.96^c^	23, 24, 26, 27	34.86, CH	2.96^c^	23, 24, 26, 27
26	176.52, C	​	​	176.45, C	​	​
27	17.29, CH_3_	1.20 s	24, 25, 26	17.14, CH_3_	1.20 days (6.9)	24, 25, 26
30	28.32, CH_3_	1.04 s	3, 5, 10, 31	27.36, CH_3_	1.13s	3, 4, 5, 31
31	15.60, CH_3_	0.85 s	3, 10, 30	20.69, CH_3_	1.10s	3, 4, 5, 30
32	24.56, CH_3_	1.40 s	8, 13, 14, 15	19.81, CH_3_	1.34s	8, 13, 14, 15
COOMe	52.07, CH_3_	3.69 s	26	51.88, CH_3_	3.69s	26

^a^ Spectra were recorded in CDCl_3_, at 600 MHz (1H) and 150 MHz (13C); chemical shifts are given in ppm; J values are in parentheses and reported in Hz; assignments were confirmed by 1H-1H-COSY, and 1H-13C-HSQC, experiments.

^b^ HMBC, correlations are from proton(s) stated to the indicated carbon.

^c^ Overlapped signal.

**FIGURE 3:**
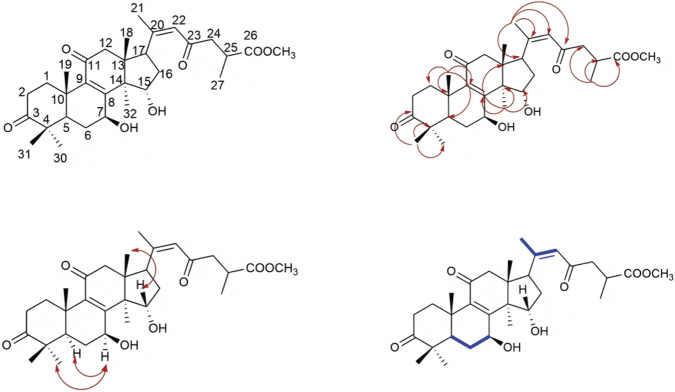
The key 2D-NMR correlations of 2.

Compound 2 is characterized as a white solid powder. HR-ESI-MS identified a peak at m/z 529.3154 [M + H]+, closely aligning with the theoretical value of m/z 529.3160, thereby suggesting a molecular formula of C_31_H_44_O_7_ and indicating a degree of unsaturation of 10. The ^13^C-NMR and HSQC spectra (refer to Table 1) revealed 31 distinct carbon signals. These include three ketone carbonyl carbons (δ_C_ 216.72, 198.62, 198.08), one ester carbon (δ_C_ 176.45), and two pairs of double bond carbons (δ_C_ 158.32, 156.35, 140.39, 124.19). Additionally, the compound comprises seven methyl carbon atoms (δ_C_ 18.97, 19.31, 21.23, 17.14, 27.36, 20.69, and 19.81), one methoxy carbon atom (δ_C_ 51.88), two oxygen-linked carbon atoms (δ_C_ 72.79 and 68.96), six methylene carbon atoms, three methine carbon atoms, and four quaternary carbon atoms. The presence of carbonyl groups and double bonds accounts for six degrees of unsaturation. The chemical shifts of the seven methyl hydrogen atoms observed in the ^1^H-NMR spectra (δ_H_ 2.11, 1.34, 1.27, 1.20, 1.13, 1.10, and 0.81) preliminarily suggest that the compound possesses a lanostane-type triterpene skeleton. The A, B, C, and D rings of this skeleton collectively contribute four degrees of unsaturation, which is consistent with the overall degree of unsaturation of 10 for the compound. In the HMBC spectrum (Figure 3), the proton H-27 (δ_H_ 1.20) exhibits correlations with carbon atoms C-26 (δ_C_ 176.45), C-25 (δ_C_ 34.86), and C-24 (δ_C_ 47.71). Additionally, the proton H-COOCH3 (δ_H_ 3.69) shows correlation with C-26, suggesting substitution of the hydrogen atom on the carboxyl group at C-26 with a methyl group. The proton H-22 (δ_H_ 6.10) correlates with carbon atoms C-17 (δ_C_ 52.12), C-20 (δ_C_ 156.35), C-21 (δ_C_ 21.23), and C-23 (δ_C_ 198.08) in the HMBC spectrum. Similarly, the proton H-18 (δ_H_ 0.81) correlates with carbon atoms C-12 (δ_C_ 50.47), C-13 (δ_C_ 48.09), and C-14 (δ_C_ 53.31). The proton H-19 (δ_H_ 1.27) correlates with carbon atoms C-1 (δ_C_ 35.44), C-10 (δ_C_ 38.07), C-5 (δ_C_ 48.72), and C-9 (δ_C_ 140.39). Furthermore, the proton H-32 (δ_H_ 1.34) exhibits correlations with carbon atoms C-13 (δ_C_ 48.09), C-14 (δ_C_ 53.31), C-8 (δ_C_ 158.32), and C-15 (δ_C_ 72.79) in the HMBC spectrum. Consequently, the planar structure of the compound is deduced as depicted in Figure 3. A literature review indicates that the compound shares structural similarities with the previously reported lanosta-8,20 (22)-dien-3,11,23-trioxo-7β,15β-diol-26-oic acid methyl ester (Ma et al., 2022), with differences observed in the spatial configurations at the 7-OH and 15-OH groups. The analysis of the 2D-NMR and ROESY spectra indicates proximal spatial arrangements between H-7 and H-30, as well as H-15 and H-18, thereby confirming the compound’s relative configuration as 7-β-OH and 15-α-OH. A search conducted in the SciFinder database revealed that this compound is novel and has not been previously documented. Consequently, the structure of compound 2 has been determined to be lanosta-8,20 (22)-dien-3,11,23-trioxo-7β,15α-diol-26-oic acid methyl ester, and it has been designated as methyl ganoderenic acid A.

Additional compounds were identified as known entities by comparing their spectral data with those available in the literature, specifically: Ganoderic acid E (Iwatsuki et al., 2003; Wang et al., 2019), Ganoderic acid D (Chen and Chen, 2020; Zhang et al., 2024), methyl ganoderate D (Chen et al., 2020; Kikuchi et al., 1986), Ganoderic acid F (Ahmad et al., 2023; Iwatsuki et al., 2003), 12β-Acetoxy-3,7,11,15,23-pentaoxo-lanost-8,20-dien-26-oic acid (Gu et al., 2016; Wei et al., 2018), Ganoderic acid H (Dongiovanni et al., 2021; Wang et al., 2019), methyl ganoderate G (Kikuchi et al., 1986; Zhong et al., 2022), and methyl ganoderate A (Lee et al., 2010; Lee et al., 2020).

### 3.2 Activity test

#### 3.2.1 Optimal oleic acid concentration in HepG2 cell model of steatosis

**FIGURE 4:**
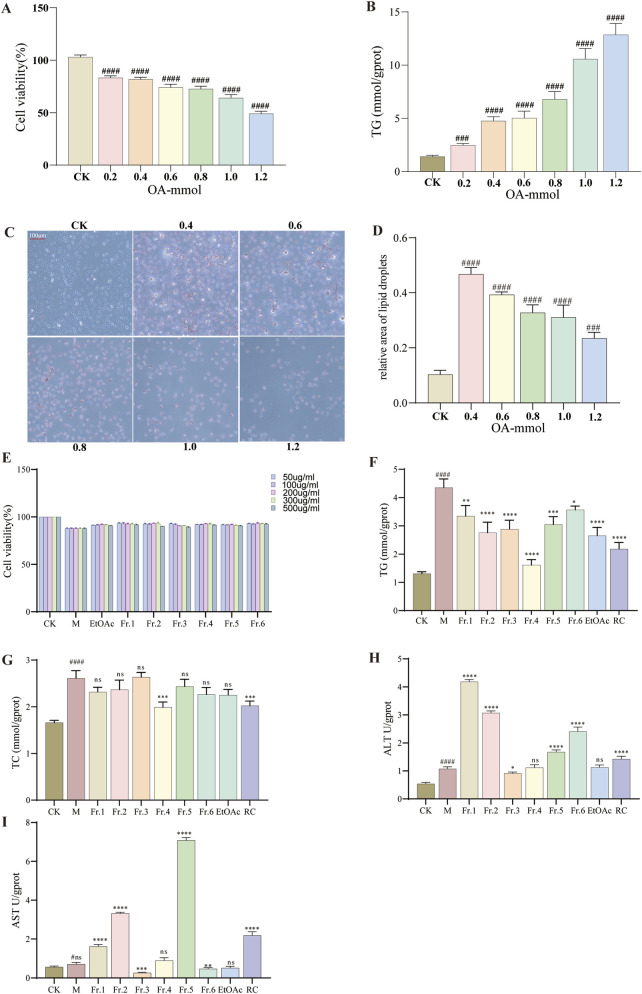
Construction of an OA-induced HepG2 cell steatosis *in vitro* model and the lipid-lowering activity of different fractions. **(A)** Cell viability of different concentrations of OA. **(B)** TG levels of different concentrations of OA. **(C)** Oil Red O staining. **(D)** Relative area of lipid droplets. **(E)** Cell viability of different concentrations of fractions. **(F)** TG levels of different fractions. **(G)** TC levels of different fractions. **(H)** ALT levels of different fractions. **(I)** AST levels of different fractions. Subsequent biological indicator studies of different fractions uniformly use a concentration of 300 μg/mL. Data are shown as mean ± SD (n = 3). (#) p < 0.05, (##) p < 0.01, (###) p < 0.001, (####) p < 0.0001 and versus non-induced cells (Control). (*) p < 0.05, (**) p < 0.01, (***) p < 0.001, (****) p < 0.0001 versus induced cells. ns: not-significant. (Model). OA: oleic acid. TG: total triglycerides. TC: total cholesterol. ALT: alanine aminotransferase. AST: aspartate aminotransferase. RC: rosuvastatin calcium.

Following a 24-h treatment of HepG2 cells with varying concentrations of OA, the effects on cell viability and intracellular TG content are depicted in Figures 4A,B. A notable decrease in cell viability and a significant increase in intracellular TG content were observed with escalating OA concentrations. At an OA concentration of 0.4 mmol/L cell viability was reduced to 80.18% (*p* < 0.001), indicating a suppression of HepG2 cell proliferation. Lipid droplet formation was assessed using Oil Red O staining, as illustrated in Figures 4C,D. Untreated HepG2 cells exhibited distinct boundaries, intact nuclear membranes, and an absence of red lipid droplets. In contrast, OA-treated cells displayed the presence of red lipid droplets, with their number markedly increasing at higher OA concentrations. Based on the results of cell viability, TG content, and Oil Red O staining, a concentration of 0.4 mmol/L was selected to induce HepG2 cells for 24 h, thereby establishing an *in vitro* model of NAFLD.

#### 3.2.2 Effects of *G. lucidum* fractions and pure compounds on cell proliferation and lipid accumulation in steatotic HepG2 cells

**FIGURE 5:**
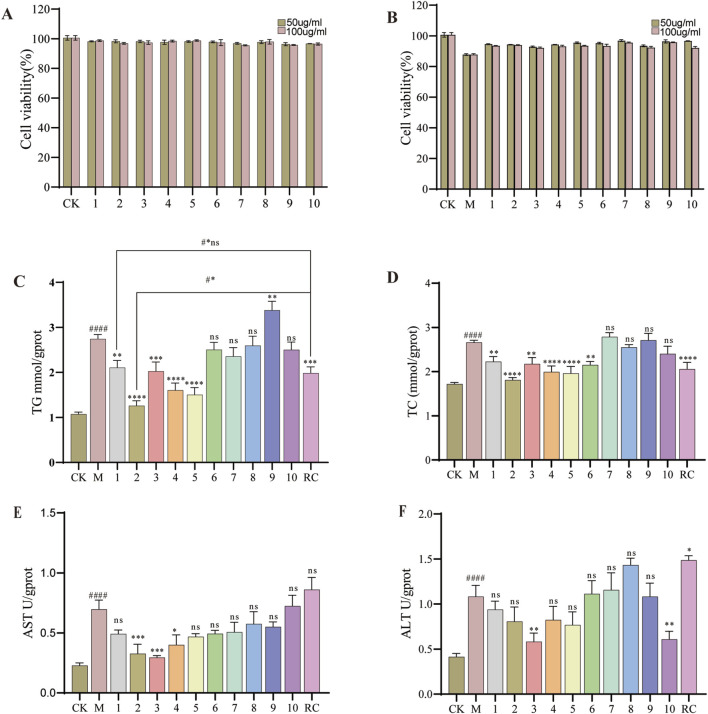
Thelipid-lowering activity of different compounds. **(A)** Effect of the compound on HepG2 cell viability. **(B)** Effects of the compound on HepG2 cell viability induced by OA. **(C)** TG levels. **(D)** TC levels. **(E)** ALT levels. **(F)** AST levels. Data are shown as mean ± SD (n = 3). (#) p < 0.05, (##) p < 0.01, (###) p < 0.001, (####) p < 0.0001 and versus non-induced cells (Control). (*) p < 0.05, (**) p < 0.01, (***) p < 0.001, (****) p < 0.0001 versus induced cells (Model). (#*) p < 0.001, #*ns: not-significant versus non-induced cells (RC). Subsequent biochemical indicator studies of pure compounds will uniformly use 100 μg/mL for research.

The survival rates of HepG2 cells exhibiting lipid degeneration, following treatment with various fractions of *G. lucidum*, are presented in Figure 4E. In comparison to the model group, the different fractions, administered at concentrations ranging from 50 to 500 μg/mL, did not exhibit significant cytotoxic effects on HepG2 cells post-model induction. Subsequently, we investigated the impact of these fractions at a concentration of 300 μg/mL on the intracellular levels of TG, TC, ALT, and AST in steatotic HepG2 cells, with the findings illustrated in Figures 4F–I. Notably, Fraction 4 significantly decreased intracellular TG and TC levels, while it did not exert a significant effect on ALT and AST levels. Consequently, Fraction 4 was further fractionated to isolate 10 distinct compounds, whose activities on steatotic HepG2 cells were subsequently assessed. The effects of the ten compounds on HepG2 cell viability are illustrated in Figure 5A. These compounds did not exhibit cytotoxicity towards HepG2 cells at either concentration tested, as indicated by a survival rate exceeding 90%. Subsequently, we investigated the influence of these compounds on the toxicity of OA-induced HepG2 cell models at two concentrations concurrently. At concentrations of 50 μg/mL and 100 μg/mL, the compounds slightly enhanced cell viability following model induction, as shown in Figure 5B. Consequently, all subsequent biochemical indicator measurements were conducted using a concentration of 100 μg/mL. The results for TG, TC, ALT, and AST levels are presented in Figures 5C–F. It was observed that compounds 1- 5 significantly reduced lipid droplet levels in the model cells (*p* < 0.05). In comparison to the positive control drug, compound 1 did not exhibit a statistically significant difference in lipid-lowering activity, (2.11 mmol/gprot vs. 1.98 mmol/gprot, *p* > 0.05). Nonetheless, compound 2 demonstrated a significant reduction in TG levels within it (1.27 mmol/gprot vs. 1.98 mmol/gprot, *p* < 0.001). Therefore, the possible mechanisms of action of compound 2 were further investigated using network pharmacology techniques.

### 3.3 Analysis of the molecular mechanism of compound 2 in the treatment of NAFLD

**FIGURE 6:**
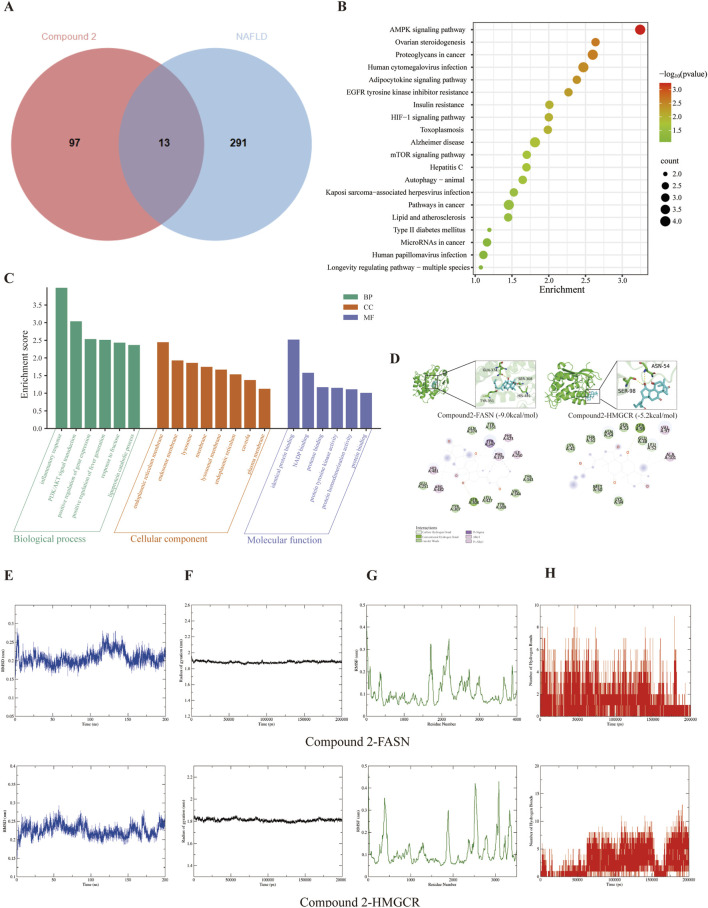
Network pharmacology, Molecular docking analysis, and Molecular dynamics simulation analysis. **(A)** Drug-target-venn diagram. **(B)** KEGG enrichment analysis. **(C)** GO analysis. **(D)** Visualization of molecular docking. **(E)** RMSD analysis; **(F)** RG analysis. **(G)** RMSF analysis. **(H)** Hydrogen bond analysis.

Compound 2, identified as Target 110, was retrieved using the Swiss Target Prediction database. Utilizing “NAFLD” as a keyword, a comprehensive search across the GenCards (Relevance score ≥ 2.59) and OMIM databases yielded 304 disease-associated targets for NAFLD. Employing Venny2.1.0, a “drug-disease” Venn diagram was constructed, revealing 13 potential therapeutic targets (see Figure 6A). Potential therapeutic targets were submitted to the DAVID database for Gene Ontology (GO) functional enrichment analysis (Figure 6C) and Kyoto Encyclopedia of Genes and Genomes (KEGG) pathway enrichment analysis (Figure 6B). The analysis revealed enrichment in 20 biological processes, 19 of which exhibited statistical significance (*p* < 0.05). These processes included the inflammatory response, lipoprotein catabolic process, and PI3K/AKT signal transduction. Additionally, six molecular functions were identified, such as NADP binding and protease binding. The analysis also highlighted ten cellular components, predominantly involving the endoplasmic reticulum membrane, endosome membrane, lysosome, and endoplasmic reticulum. KEGG pathway enrichment analysis identified 20 signaling pathways, including the AMPK signaling pathway, ovarian steroidogenesis, and adipocytokine signaling pathway. Notably, the targets associated with the AMPK signaling pathway were FASN and HMGCR.

### 3.4 Molecular docking and molecular dynamics simulation

Consequently, molecular docking was employed to determine the binding energies of Compound 2 with these targets, and the results were visualized (Figure 6D). Following the execution of molecular docking analysis utilizing AutoDock software, it was determined that compound 2 exhibited binding energies of −9.0 kcal/mol and −5.2 kcal/mol with the targets FASN and HMGCR, respectively, suggesting a strong affinity. The binding interactions of compound 2 with these targets are illustrated in Figure 6D. Specifically, in the case of FASN, compound 2 engages in a hydrogen bond interaction with SER308 and establishes hydrophobic interactions with HIS481, ARG482, PHE370, ILE250, and PHE423. Similarly, the interaction of compound 2 with HMGCR is characterized by a hydrogen bond with SER98 and hydrophobic interactions with VAL97 and ALA101. These interactions underpin the binding of small molecules to protein targets. While molecular docking provides insights into the static binding conformation of the small molecule to the protein, we further validated the dynamic nature of the interaction between compound 2 and the target proteins through molecular dynamics simulation experiments. To further substantiate the dynamic interaction of compound 2 with FASN and HMGCR, we performed molecular dynamics simulations. The outcomes are illustrated in Figures 6E–H. In the simulation of compound 2 with FASN, the root-mean-square deviation (RMSD) values exhibited slight fluctuations over a 200 ns timeframe, suggesting stable conformational binding. Furthermore, the radius of gyration (Rg), which assesses protein compactness, also displayed minimal fluctuation over the same period, indicating increased protein compactness following ligand binding. The residue fluctuation analysis (RMSF) demonstrated that upon binding with the protein ligand, the small molecule maintained relatively low RMSF values, signifying stable binding. Hydrogen bond analysis revealed that over the 200 ns duration, the small molecule consistently formed a stable number of hydrogen bonds with the protein ligand. Collectively, these findings indicate that compound 2 exhibits stable binding to FASN. Similarly, the molecular dynamics simulation results for compound 2 with HMGCR also confirm a stable interaction between the two entities.

## 4 Discussion

This study employed an activity-guided isolation approach to systematically identify lipid-lowering compounds from the traditional medicinal fungus Ganoderma, utilizing TG levels in OA-induced HepG2 cells as an evaluation metric. Through a series of chromatographic techniques, a total of 10 compounds were successfully isolated. The structural elucidation of these compounds was accomplished using mass spectrometry, NMR, IR, and UV spectroscopy, revealing two previously unreported lanostane-type compounds (1–2) and eight known compounds (3–10). The lipid-lowering efficacy of these compounds was assessed using an *in vitro* model of steatotic cells, with measurements of TG, TC, ALT, and AST levels following intervention. It was observed that compounds 1-5 significantly reduced TG levels in the model group. The structural differences in lipid-lowering activity between these five compounds and compounds 6–10 may be attributed to variations in the functional groups attached at the C-7 and C-15 positions. In comparison with the positive control drug, compound 2 exhibited superior lipid-lowering activity (*p* < 0.01), whereas the lipid-lowering activity of compound 1 did not differ significantly from the positive control (*p* > 0.05). Consequently, we conducted an in-depth investigation into the potential mechanisms of compound 2 utilizing network pharmacology, molecular docking, and molecular dynamics simulation methodologies. Within the network pharmacology analysis, we identified 13 potential overlapping targets by examining the targets of compound 2 alongside those associated with NAFLD. GO and KEGG enrichment analyses indicated that compound 2 might influence lipid metabolism via the AMP-activated protein kinase (AMPK) signaling pathway. As a pivotal regulator of cellular energy metabolism, AMPK activation can concurrently inhibit FASN-mediated fatty acid synthesis and HMGCR-mediated cholesterol biosynthesis. The analysis identified FASN and HMGCR as key proteins enriched among the common targets of methyl ganoderenic acid A and NAFLD. FASN serves as a pivotal rate-limiting enzyme in the synthesis of fatty acids within the body, and its upregulation directly contributes to the excessive accumulation of triglycerides in hepatocytes (Hu et al., 2021). Similarly, HMGCR is a well-established rate-limiting enzyme in the cholesterol biosynthesis pathway and constitutes the primary target of statin lipid-lowering therapies in clinical settings (Min et al., 2012).

Subsequent molecular docking analyses have demonstrated that methyl ganoderenic acid A can establish complementary structures within the active sites of two key targets, exhibiting strong binding affinity. Further molecular dynamics simulations revealed that the complexes formed between methyl ganoderenic acid A and these targets maintained structural stability over a 200 ns simulation period. Throughout the simulation, hydrogen bonds and hydrophobic interactions involving critical amino acid residues were consistently observed, theoretically affirming the reliability of their binding interactions. A comprehensive analysis suggests that methyl ganoderenic acid A may possess lipid-lowering properties through a multi-target synergistic mechanism. Specifically, it appears to attenuate endogenous cholesterol synthesis by inhibiting HMGCR and reduce excessive fatty acid production by modulating the expression of FASN. This multi-target approach aligns with the traditional holistic regulatory characteristics of Ganoderma, offering a theoretical foundation for its development as a novel lipid-lowering agent. However, this study primarily utilized computational methods to explore these possibilities, necessitating further in-depth investigation. Notably, this study is the first to document the lipid-lowering activity of a specific compound within Ganoderma and elucidate its potential mechanism, thereby providing new scientific insights into its medicinal value and identifying a promising lead compound for the treatment of NAFLD. Future research will concentrate on *in vivo* animal studies to validate its efficacy and further optimize its structure to enhance its activity and selectivity.

## 5 Conclusion

In conclusion, a comprehensive phytochemical investigation was performed on the ethyl acetate extract of *G. lucidum* fruiting bodies, leading to the successful isolation of ten triterpenoid compounds, including two novel entities and eight known analogs. Structurally, compounds 1 and 2 are classified as C_30_-type triterpenoids. The lipid-lowering potential of all isolated compounds (1–10) was assessed using a HepG2 cell high-lipid model. Notably, compound 2 demonstrated significant lipid-lowering activity in comparison to the model group. Through the application of network pharmacology, molecular docking, and molecular dynamics simulations, it was identified that compound 2 may interact with targets such as FASN and HMGCR, and potentially influence the AMPK signaling pathway. These findings offer valuable insights for the pharmacological exploration of *G. lucidum* and suggest that this class of compounds holds promise as potential therapeutic agents for mitigating NAFLD.

## Data Availability

The original contributions presented in the study are publicly available. This data can be found here: https://doi.org/10.6084/m9.figshare.31073521

## References

[B1] Ahmad M. F. Ahmad F. A. Zeyaullah M. Alsayegh A. A. Mahmood S. E. AlShahrani A. M. (2023). Ganoderma lucidum: Novel insight into hepatoprotective potential with mechanisms of action. Nutrients 15 (8), 1874. 10.3390/nu15081874 37111092 PMC10146730

[B2] Chen D. H. Chen W. K. D. (2020). Determination of ganoderic acids in triterpenoid constituents of Ganoderma tsugae. J. Food Drug Anal. 11 (3). 10.38212/2224-6614.2699

[B3] Chen M. Xiao D. Liu W. Song Y. Zou B. Li L. (2020). Intake of Ganoderma lucidum polysaccharides reverses the disturbed gut microbiota and metabolism in type 2 diabetic rats. Int. J. Biol. Macromol. 155, 890–902. 10.1016/j.ijbiomac.2019.11.047 31712153

[B4] Dongiovanni P. Meroni M. Longo M. Fargion S. Fracanzani A. L. (2021). Genetics, immunity and nutrition boost the switching from NASH to HCC. Biomedicines 9 (11), 1524. 10.3390/biomedicines9111524 34829753 PMC8614742

[B5] Gu J. Yao M. Yao D. Wang L. Yang X. Yao D. (2016). Nonalcoholic lipid accumulation and hepatocyte malignant transformation. J. Clin. Transl. Hepatol. 4 (2), 123–130. 10.14218/JCTH.2016.00010 27350942 PMC4913080

[B6] Hu Y. He W. Huang Y. Xiang H. Guo J. Che Y. (2021). Fatty acid synthase–suppressor screening identifies sorting nexin 8 as a therapeutic target for NAFLD. Hepatology 74 (5), 2508–2525. 10.1002/hep.32045 34231239

[B7] Iwatsuki K. Akihisa T. Tokuda H. Ukiya M. Oshikubo M. Kimura Y. (2003). Lucidenic acids P and Q, methyl lucidenate P, and other triterpenoids from the fungus *Ganoderma lucidum* and their inhibitory effects on Epstein−Barr virus activation. J. Nat. Prod. 66 (12), 1582–1585. 10.1021/np0302293 14695801

[B8] Kikuchi T. Kanomi S. Murai Y. Kadota S. Tsubono K. Ogita Z. I. (1986). Constituents of the fungus Ganoderma lucidum (Fr.) Karst. II structures of ganoderic acids F, G, and H, lucidenic acids D2 and E2, and related compounds. Chem. Pharm. Bull. (Tokyo) 34 (10), 4018–4029. 10.1248/cpb.34.4018

[B9] Kubota T. Asaka Y. Miura I. Mori H. (1982). Structures of ganoderic acid A and B, two new lanostane type bitter triterpenes from *Ganoderma lucidum* (FR.) K ARST. Helv. Chim. Acta 65 (2), 611–619. 10.1002/hlca.19820650221

[B10] Lagrutta L. C. Montero-Villegas S. Layerenza J. P. Sisti M. S. García De Bravo M. M. Ves-Losada A. (2017). Reversible nuclear-lipid-droplet morphology induced by oleic acid: a link to cellular-lipid metabolism. PLOS ONE 12 (1), e0170608. 10.1371/journal.pone.0170608 28125673 PMC5268491

[B11] Lee I. Seo J. Kim J. Kim H. Youn U. Lee J. (2010). Lanostane triterpenes from the fruiting bodies of *Ganoderma lucidum* and their inhibitory effects on adipocyte differentiation in 3T3-L1 cells. J. Nat. Prod. 73 (2), 172–176. 10.1021/np900578h 20039640

[B12] Lee H. A. Cho J. H. Afinanisa Q. An G. H. Han J. G. Kang H. J. (2020). Ganoderma lucidum extract reduces insulin resistance by enhancing AMPK activation in high-fat diet-induced Obese mice. Nutrients 12 (11), 3338. 10.3390/nu12113338 33142995 PMC7693844

[B13] Li Q. Chang Y. He Z. Chen L. Zhou X. (2019). Immunomodulatory activity of *Ganoderma lucidum* immunomodulatory protein *via* PI3K/Akt and MAPK signaling pathways in RAW264.7 cells. J. Cell Physiol. 234 (12), 23337–23348. 10.1002/jcp.28901 31148200

[B14] Ma Q. Zhang S. Yang L. Xie Q. Dai H. Yu Z. (2022). Lanostane triterpenoids and ergostane steroids from Ganoderma luteomarginatum and their cytotoxicity. Molecules 27 (20), 6989. 10.3390/molecules27206989 36296582 PMC9611895

[B15] Min H. K. Kapoor A. Fuchs M. Mirshahi F. Zhou H. Maher J. (2012). Increased hepatic synthesis and dysregulation of cholesterol metabolism is associated with the severity of nonalcoholic fatty liver disease. Cell Metab. 15 (5), 665–674. 10.1016/j.cmet.2012.04.004 22560219 PMC3361911

[B16] Soares A. De Sá-Nakanishi A. Bracht A. Da Costa S. Koehnlein E. De Souza C. (2013). Hepatoprotective effects of mushrooms. Molecules 18 (7), 7609–7630. 10.3390/molecules18077609 23884116 PMC6270077

[B17] Wang C. Liu X. Lian C. Ke J. Liu J. (2019). Triterpenes and aromatic meroterpenoids with antioxidant activity and neuroprotective effects from Ganoderma lucidum. Molecules 24 (23), 4353. 10.3390/molecules24234353 31795252 PMC6930543

[B18] Wei J. C. Wang A. H. Wei Y. L. Huo X. K. Tian X. G. Feng L. (2018). Chemical characteristics of the fungus *Ganoderma lucidum* and their inhibitory effects on acetylcholinesterase. J. Asian Nat. Prod. Res. 20 (10), 992–1001. 10.1080/10286020.2017.1367770 28944681

[B19] Wu P. Zhang C. Yin Y. Zhang X. Li Q. Yuan L. (2024). Bioactivities and industrial standardization status of Ganoderma lucidum: a comprehensive review. Heliyon 10 (19), e36987. 10.1016/j.heliyon.2024.e36987 39435114 PMC11492437

[B20] Zhang L. Shi P. Jin P. Chen Z. Hu B. Cao C. (2024). Ganodermanontriol regulates tumor-associated M2 macrophage polarization in gastric cancer. Aging 16 (2), 1390–1398. 10.18632/aging.205434 38244580 PMC10866403

[B21] Zhong B. Li F. L. Zhao J. Y. Fu Y. Peng C. (2022). Sporoderm-broken spore powder of Ganoderma lucidum ameliorate obesity and inflammation process in high-fat diet-induced obese mice. Food Nutr. Res. 66, 66. 10.29219/fnr.v66.8745 36340916 PMC9602201

