# Supplementary material for: A new lanostane-type triterpene with lipid-lowering activity from Ganoderma lucidum and an additional analogue
Source: Front Chem. 2026 Jan 21;13:1726447. doi: 10.3389/fchem.2025.1726447 (PMC12867892; doi:10.3389/fchem.2025.1726447)
Supplement: Supplementary file 1 [file DataSheet1.pdf]

Supplementary Information

A new lanostane-type triterpene with lipid-lowering activity from Ganoderma lucidum and an additional analogue

Xuesheng Hu[1,#], Lu Han[1,#], Yiying Tan[1], Peitong Wu[1], Yajing Li[1], Wanjie Liu[1], Jiaming Shen[1], Yishan Li[1], Ming Yang[1], Chunnan Li[1,*], Jiaming Sun[1,*]

1. Jilin Ginseng Academy, Changchun University of Chinese Medicine, Changchun 130117, China.
* Correspondence: sun_jiaming2000@163.com (J.S.); lcn1013@163.com (C. L.)
#: These authors contributed equally to this work and share their first authorship

Table of contents

Figures	Content
**S1**	
**S2**	^{1}H NMR spectrum of Compound **1** in $CDCl_3$
**S3**	^{13}C NMR spectrum of Compound **1** in $CDCl_3$
**S4**	HSQC spectrum of Compound **1** in $CDCl_3$
**S5**	^{1}H-^{1}H COSY spectrum of Compound **1** in $CDCl_3$
**S6**	HMBC spectrum of Compound **1** in $CDCl_3$
**S7**	ROESY spectrum of Compound **1** in $CDCl_3$
**S8**	IR spectrum of Compound 1 in $CDCl_3$
**S9**	^{1}H NMR spectrum of Compound **2** in $CDCl_3$
**S10**	^{13}C NMR spectrum of Compound **2** in $CDCl_3$
**S11**	HSQC spectrum of Compound **2** in $CDCl_3$
**S12**	^{1}H-^{1}H COSY spectrum of Compound **2** in $CDCl_3$
**S13**	HMBC spectrum of Compound **2** in $CDCl_3$
**S14**	ROESY spectrum of Compound **2** in $CDCl_3$
**S15**	IR spectrum of Compound **2** in $CDCl_3$

Extraction and isolation

The dried fruiting bodies (5 kg) were pulverized and subjected to extraction using 95% ethanol (5 L), 70% ethanol (5 L), and purified water (5 L) under conditions of heating and reflux for 3 hours, respectively. The resulting filtrates were combined to yield a crude extract weighing 310 g (yield 6.2%). This crude extract was then suspended in warm water and extracted with EtOAc (v/v 1:1) until the solution became colorless to obtain the EtOAc extract (80.5 g). The EtOAc extract was subjected to column chromatography on silica gel with gradient elution of PE-EtOAc（v/v 100：1，90：10，70：30，50：50）and CH_2Cl_2-MeOH (v/v 100：0，40：1，20：1，10：1，5：1，1：1，0：1）to give six fractions. Fr.4 (20.3 g) was subjected to silica gel with gradient elution (v/v 100:1, 80:1, 60:1, 40:1, 20:1, 10:1, 1:1), yielding three sub-fractions, designated Fr.4.1 to Fr.4.3. Fraction 4.1 (6.5 g) was further processed using MCI column chromatography with MeOH-H_2O gradient ranging from 40% to 100%, resulting in six sub-fractions, Fr.4.1.1 to Fr.4.1.6. Fraction 4.1.4 (3.3 g) was subjected to Sephadex LH-20 column chromatography with methanol as the eluent, producing four sub-fractions, Fr.4.1.4.1 to Fr.4.1.4.4. Fraction 4.1.4.3 (2.7 g) was then processed using ODS-A chromatography, yielding four sub-fractions, Fr.4.1.4.3.1 to Fr.4.1.4.3.4. Fraction 4.1.4.3.3 (1.5 g) underwent silica gel with CH_2Cl_2-MeOH (v/v 80:1, 60:1, 40:1, 20:1, 1:1), resulting in five sub-fractions, Fr.4.1.4.3.3.1 to Fr.4.1.4.3.3.5. Finally, Fr.4.1.4.3.3.1 (75 mg) was subjected to semi-preparative HPLC using a mobile phase of 61% MeOH/H_2O at a flow rate of 3 mL/min, ultimately isolating compound 1 (1.2mg,1.6%). The fractions Fr.4.1.4.3.3.3 (110 mg), Fr.4.1.4.3.3.4 (245 mg), Fr.4.1.4.3.3.5 (330 mg), and Fr.4.1.4.3.4 (68 mg) were subjected to semi-preparative HPLC under specified conditions to isolate various compounds. Specifically, Fr.4.1.4.3.3.3 was processed using a mobile phase of 68% MeOH/H_2O at a flow rate of 3 mL/min, resulting in the isolation of compound 2 (6.0 mg,5.4%) and compound 3 (8.9 mg,8%). Similarly, Fr.4.1.4.3.3.4 was treated with a 63% MeOH/H2O at the same flow rate, yielding compound 4 (6.2 mg,2.5%) and compound 5 (7.9 mg,3.2%). Fr.4.1.4.3.3.5 was processed using a 65% MeOH/H_2O, yielding compound 6 (82 mg,24.8%), compound 7 (20.8 mg,6.3%), and compound 8 (5.8

mg,1.8%). Lastly, Fr.4.1.4.3.4 was treated with a 62% MeOH/H_2O, resulting in the isolation of compound 9 (5.8 mg,8.5%) and compound 10 (10.0 mg,14.7%).

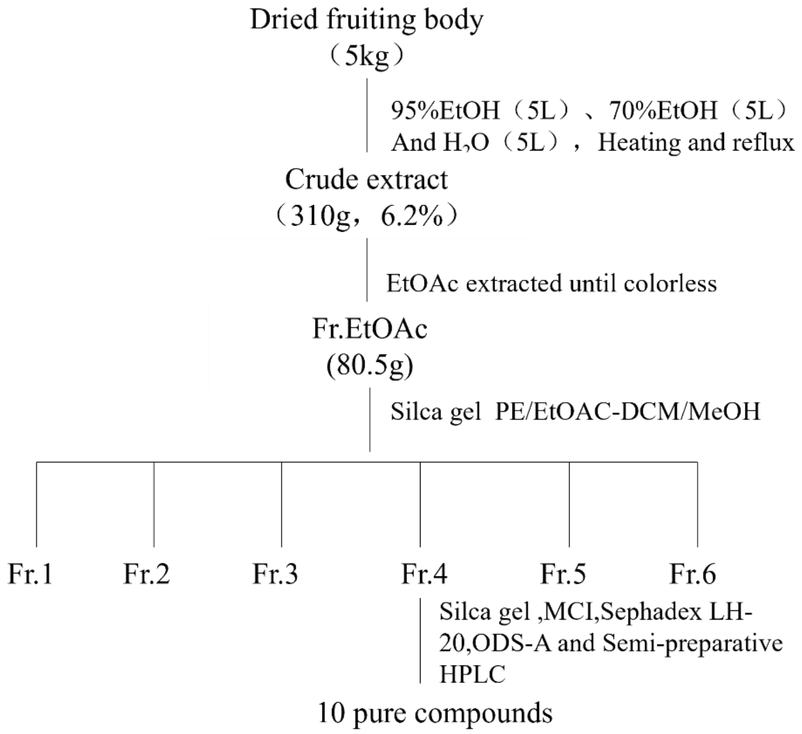

Figure S1.Extraction and isolation steps

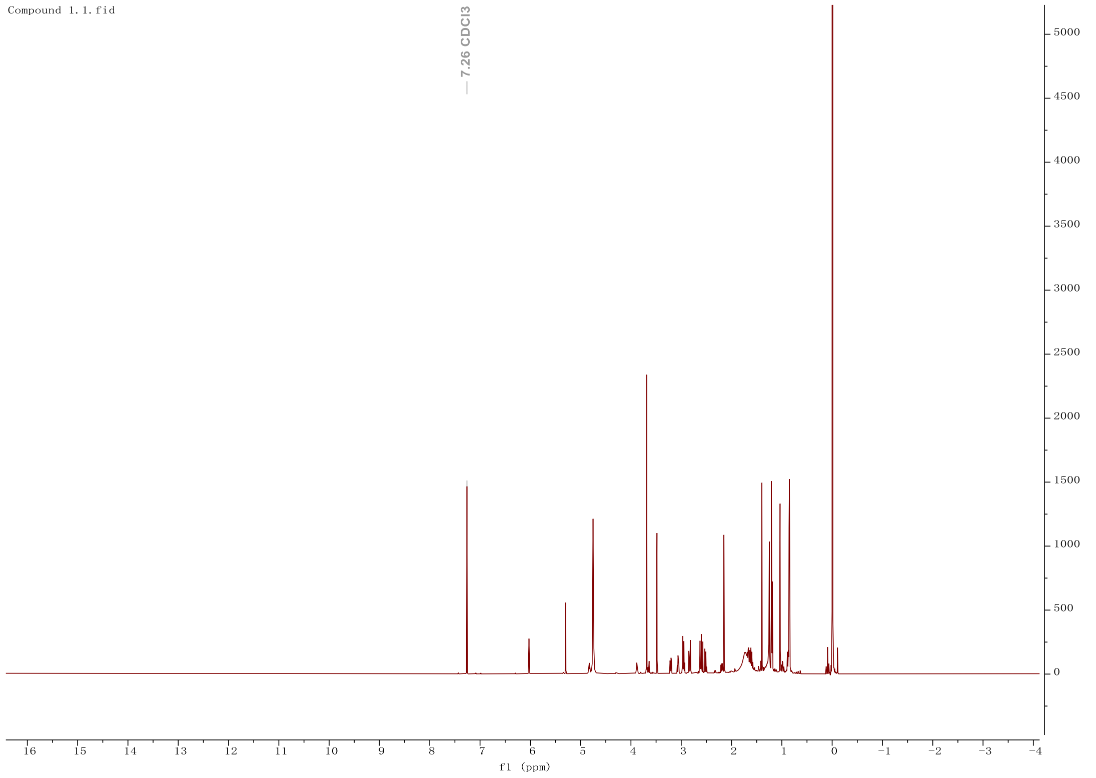

Figure S2 ^{1}H NMR spectrum of Compound **1** in $CDCl_3$.

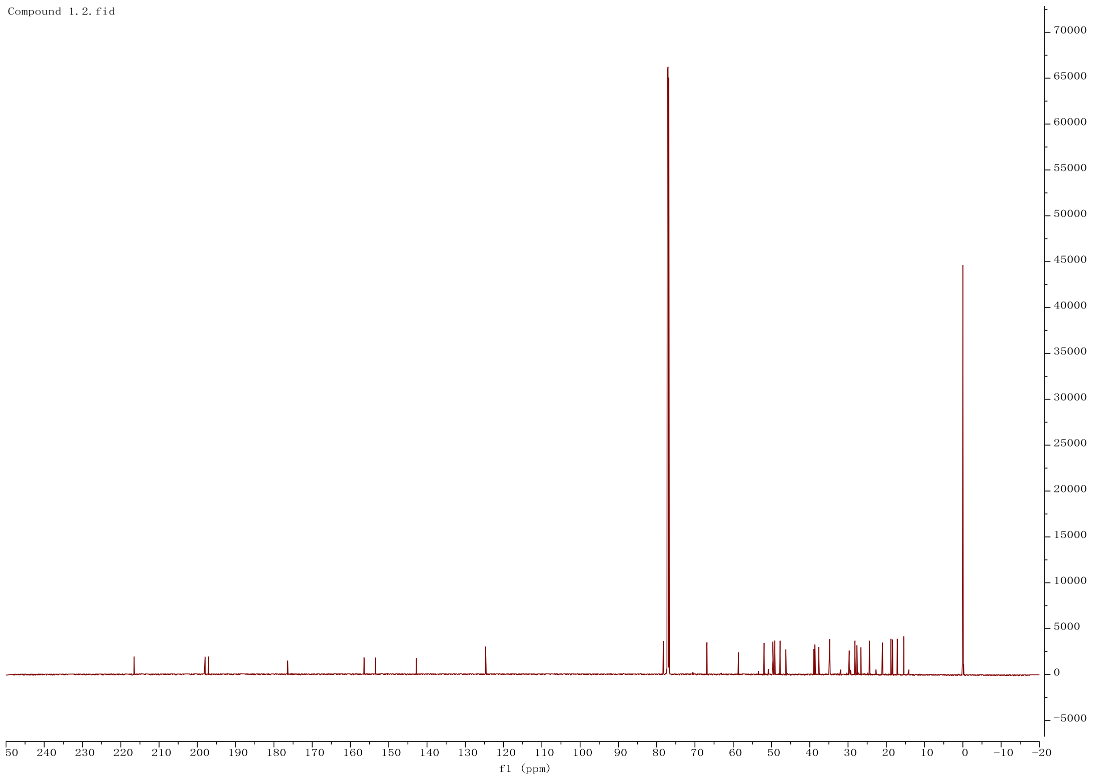

Figure S3 ^{13}C NMR spectrum of Compound **1** in $CDCl_3$.

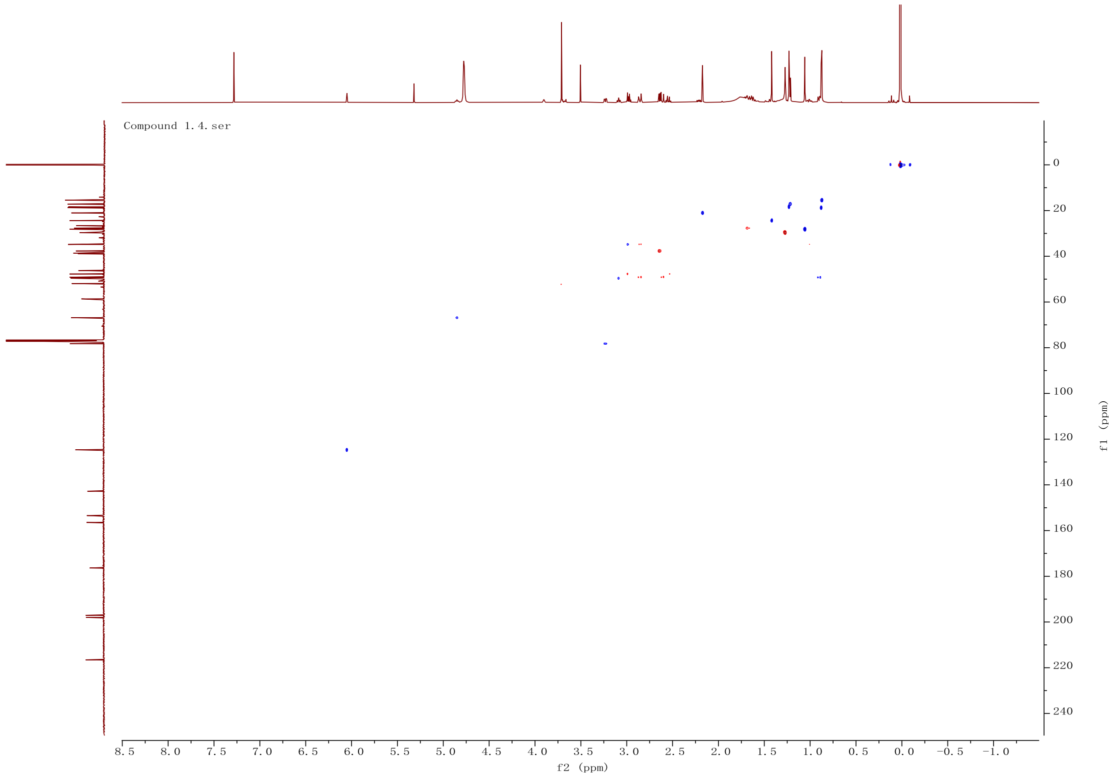

Figure S4 HSQC spectrum of Compound **1** in $CDCl_3$.

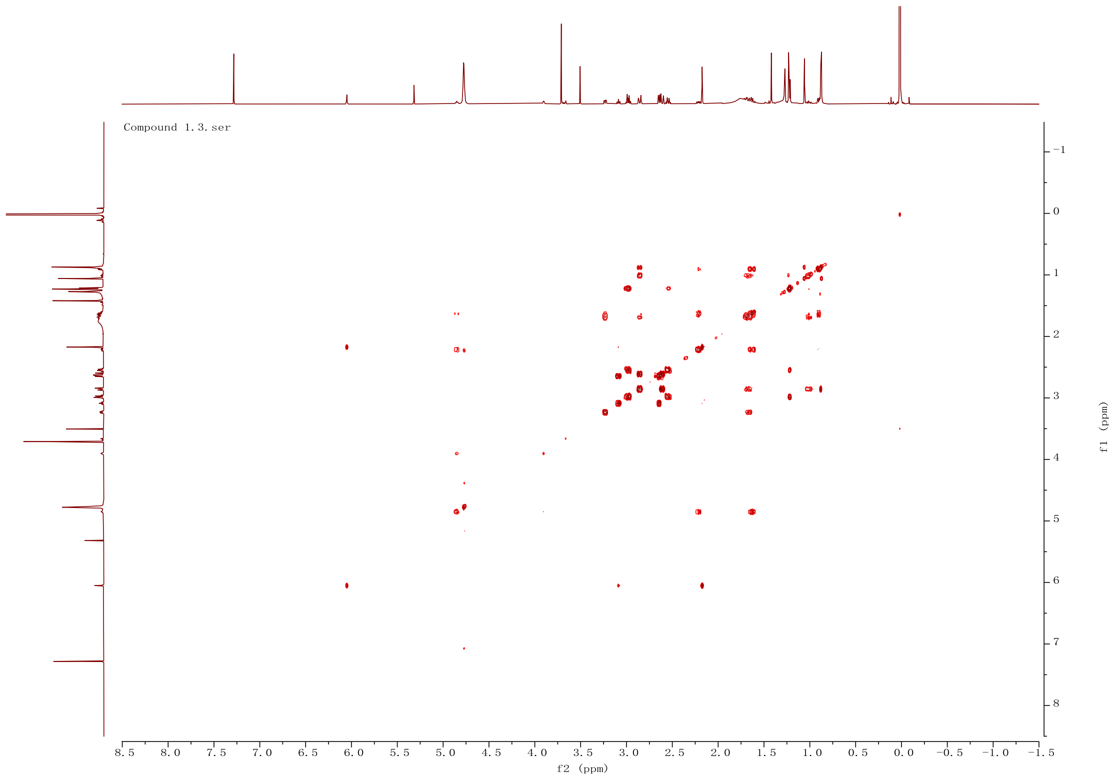

Figure S5 ^{1}H-^{1}H COSY spectrum of Compound **1** in $CDCl_3$.

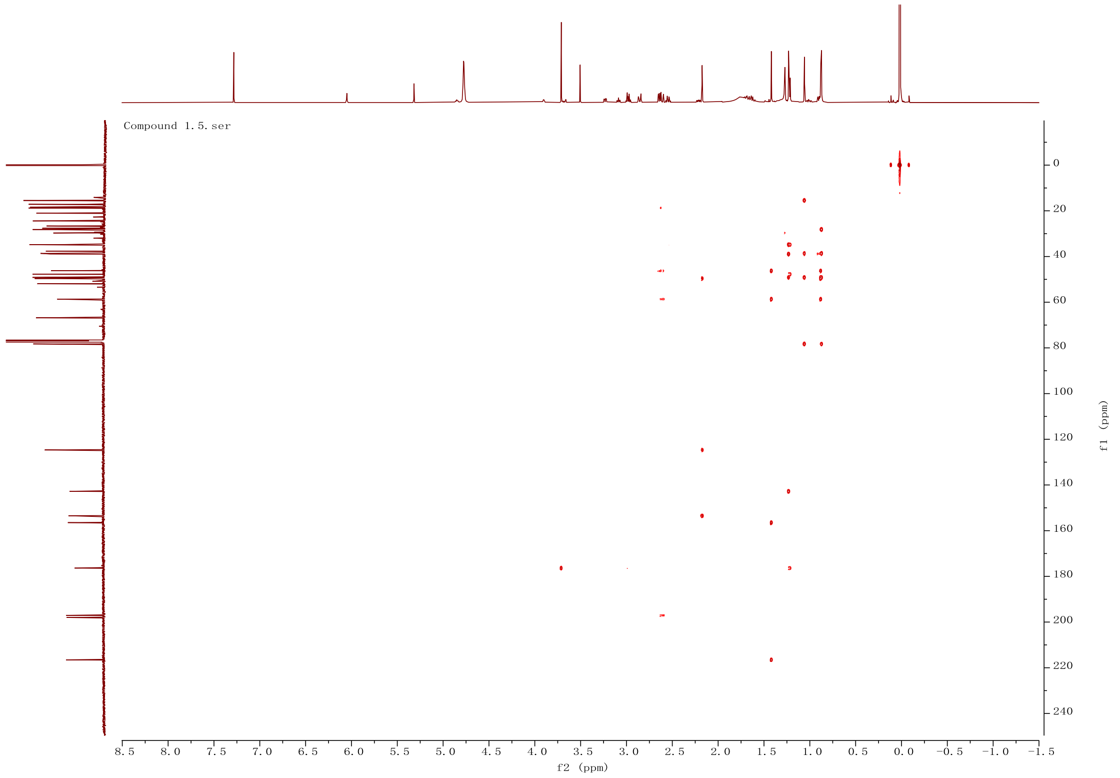

Figure S6 HMBC spectrum of Compound **1** in $CDCl_3$.

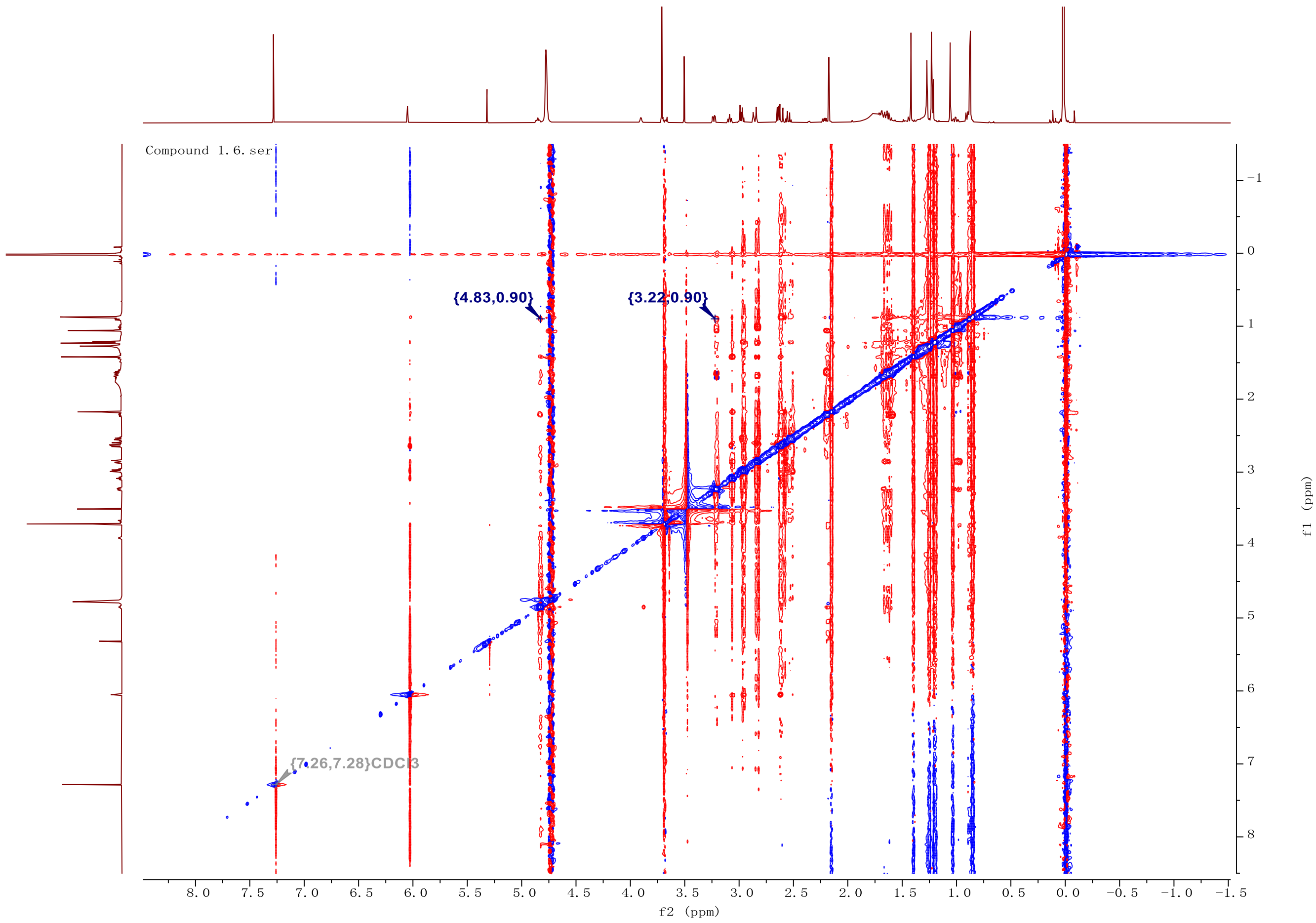

Figure S7 ROESY spectrum of Compound **1** in $CDCl_3$.

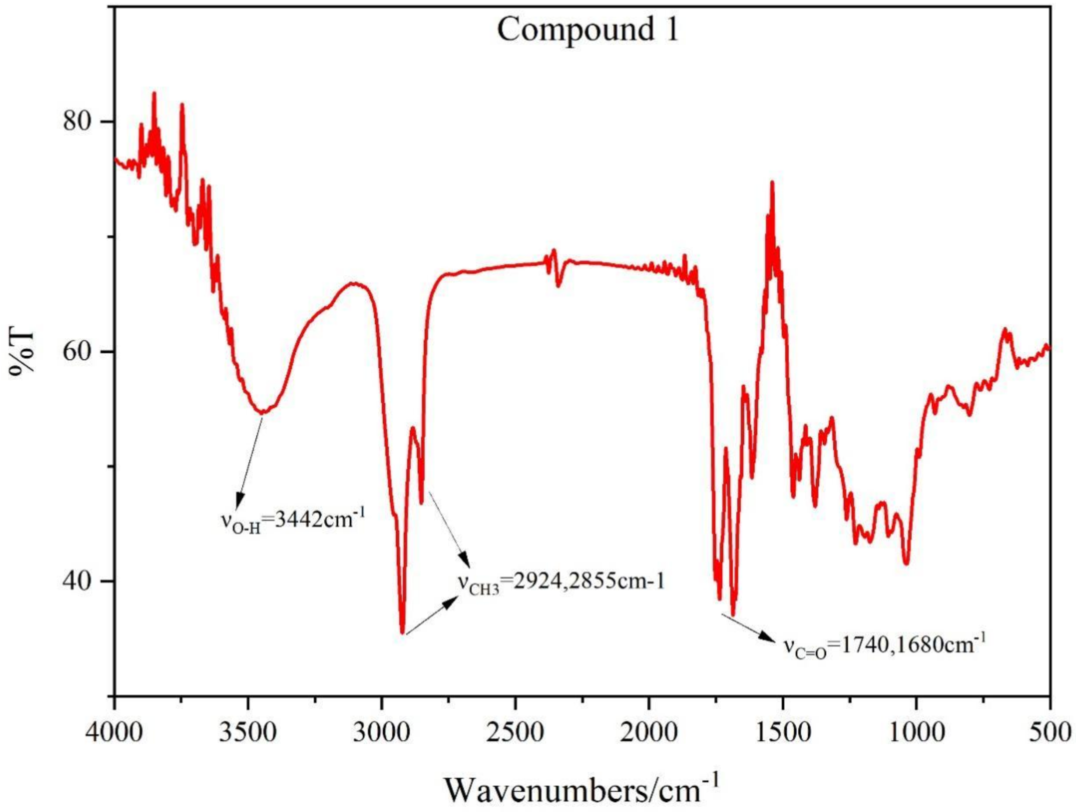

Figure S8 IR spectrum of Compound **1**

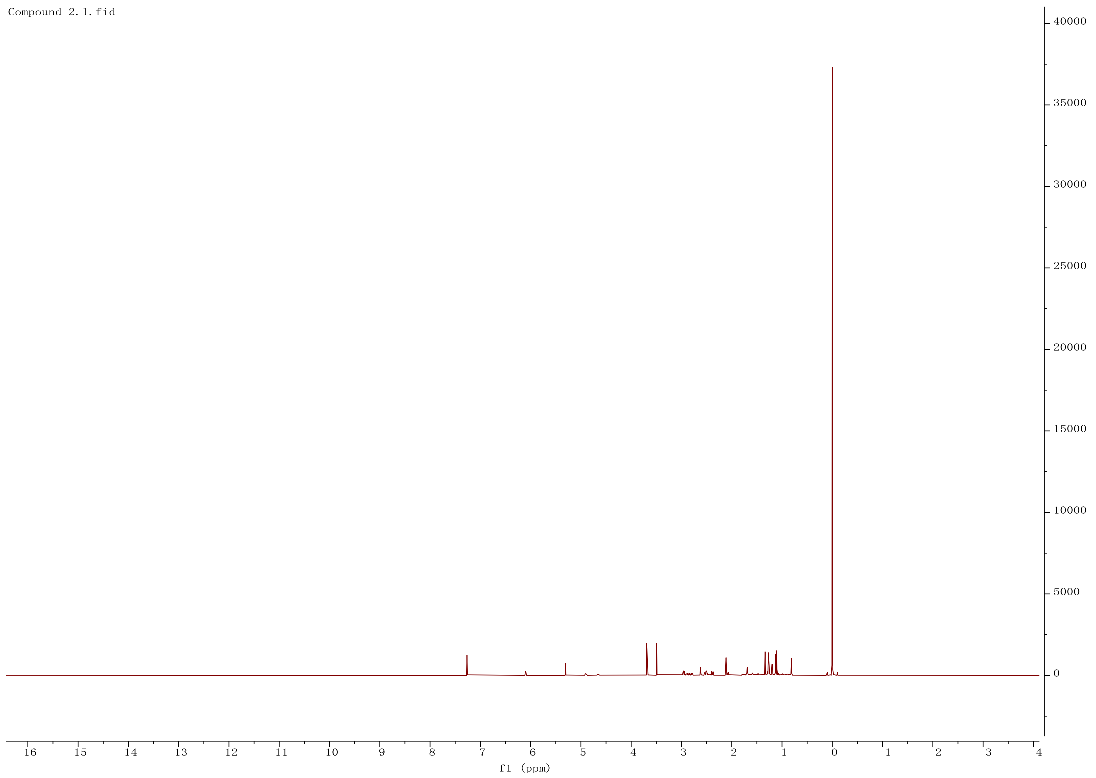

Figure S9 ^{1}H NMR spectrum of Compound **2** in $CDCl_3$.

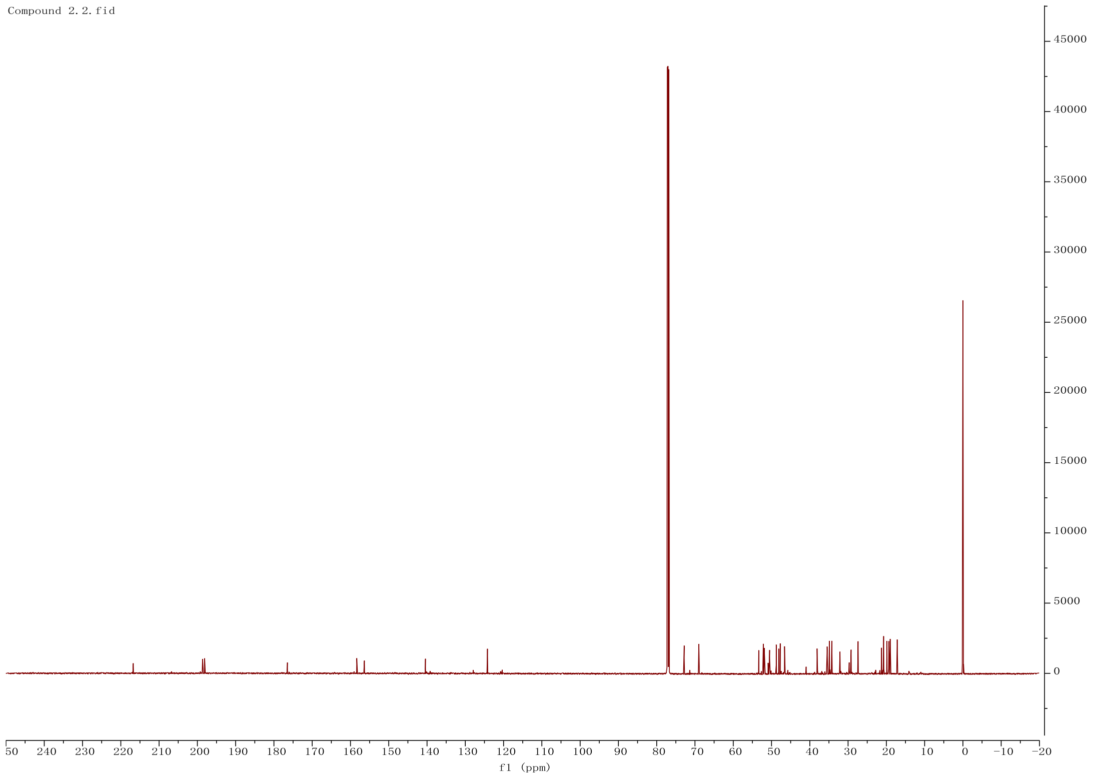

Figure S10 ^{13}C NMR spectrum of Compound **2** in $CDCl_3$.

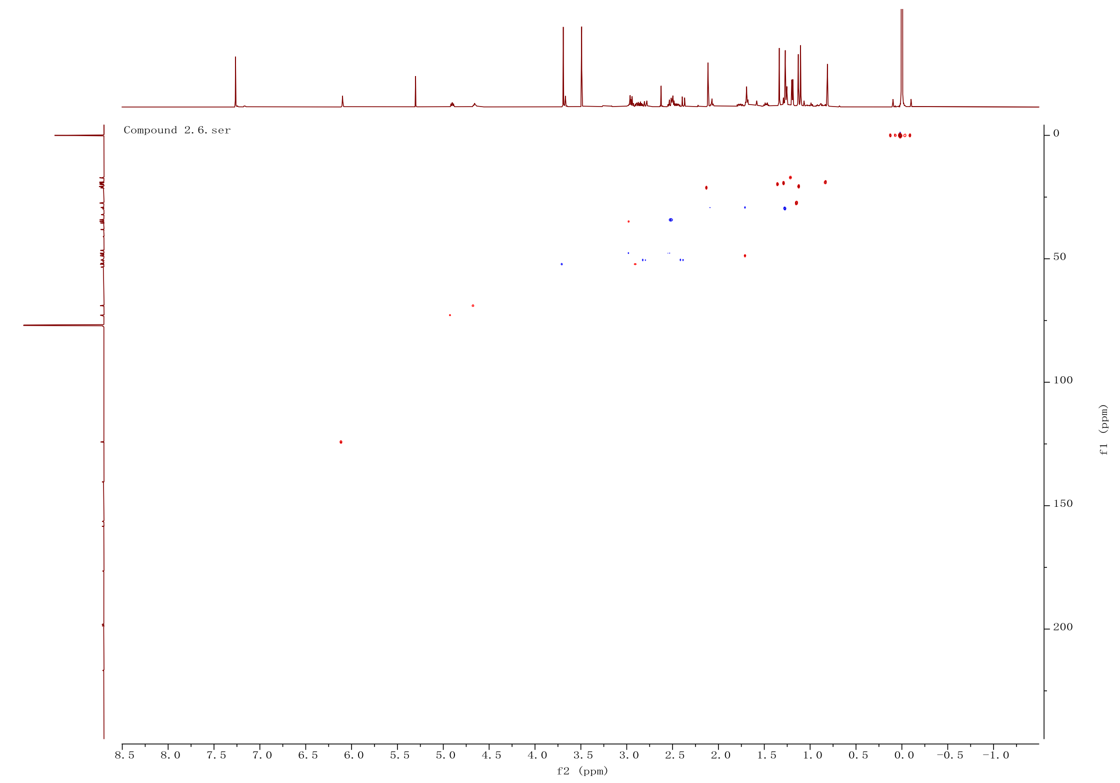

Figure S11 HSQC spectrum of Compound **2** in $CDCl_3$.

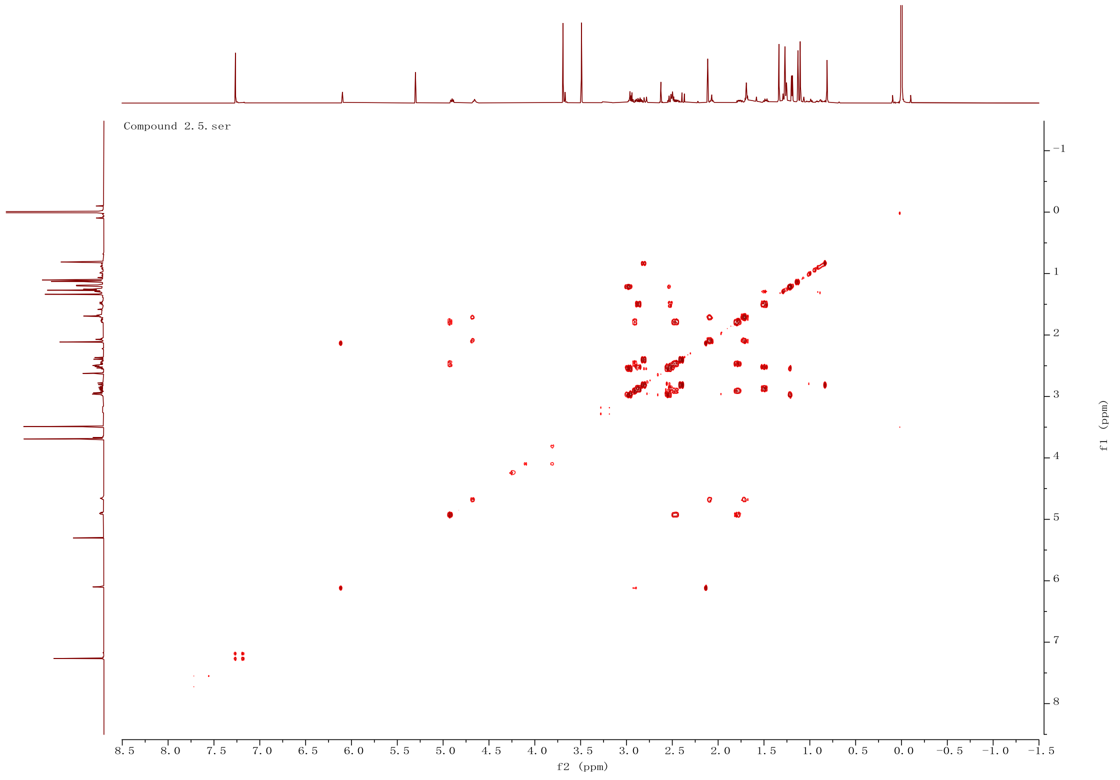

Figure S12 ^{1}H-^{1}H COSY spectrum of Compound **2** in $CDCl_3$.

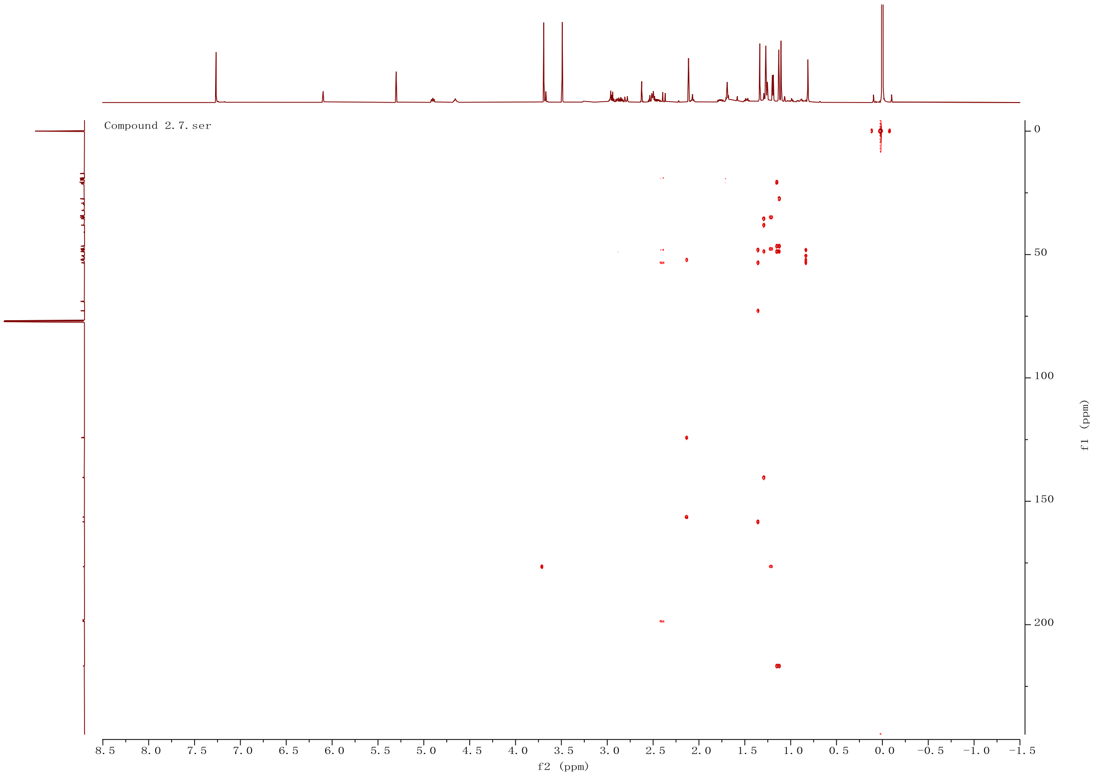

Figure S13 HMBC spectrum of Compound **2** in $CDCl_3$.

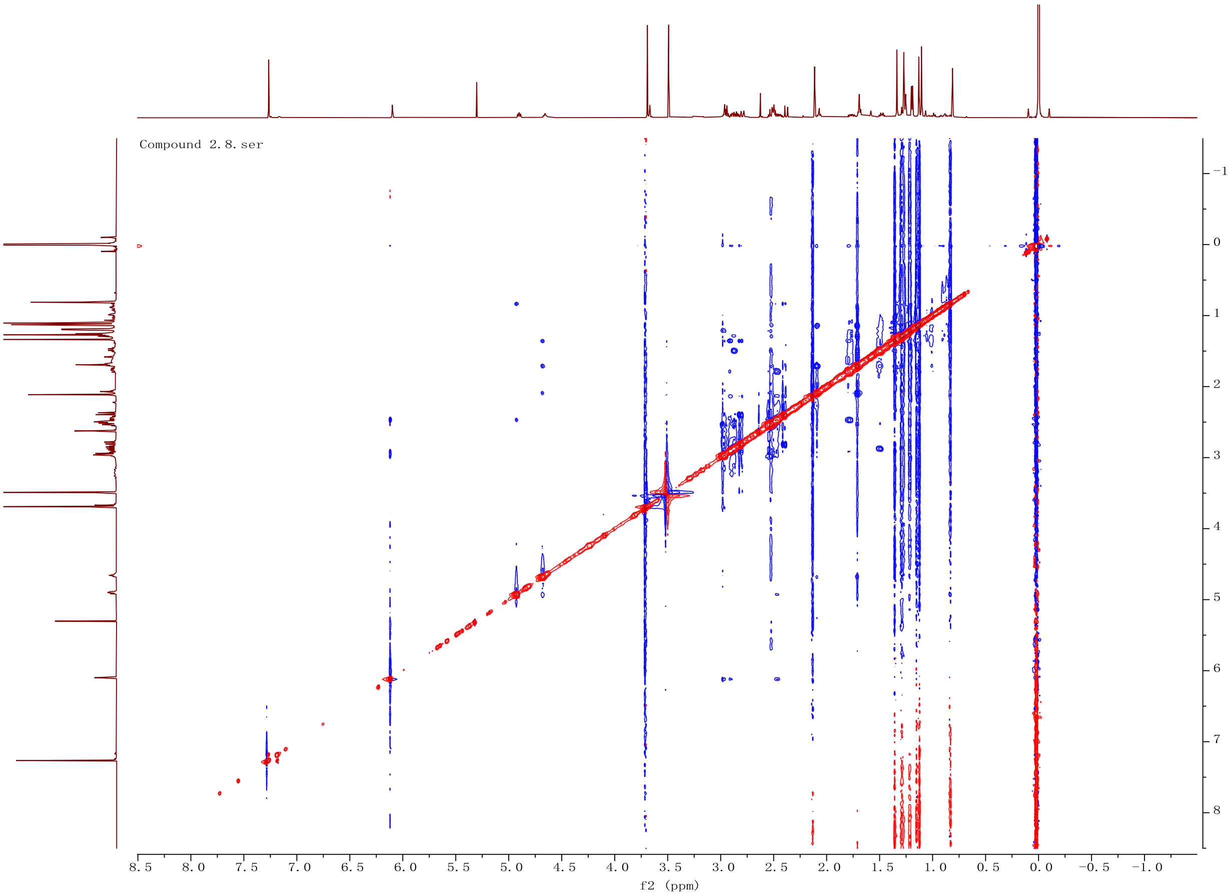

Figure S14 ROESY spectrum of Compound 2 in $CDCl_3$

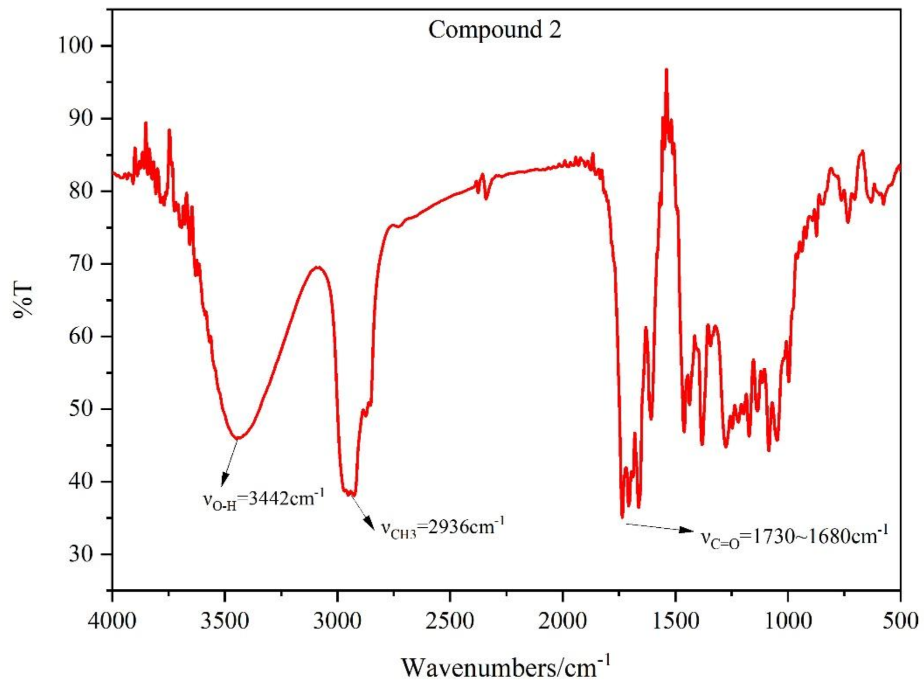

Figure S15 IR spectrum of Compound **2**
